# Evaluation of antibacterial activity of five biocides and the synergistic effect of biocide/EDTA combinations on biofilm-producing and non-producing *Stenotrophomonas maltophilia* strains isolated from clinical specimens in Iran

**DOI:** 10.1186/s12866-022-02664-1

**Published:** 2022-10-21

**Authors:** Raana Kazemzadeh Anari, Farhad Nikkhahi, Amir Javadi, Mehdi Bakht, Mohammad Rostamani, Fatemeh Zeynali Kelishomi, Safar Ali Alizadeh

**Affiliations:** 1 Medical Microbiology Research Center, Qazvin University of Medical Sciences, Qazvin, Iran; 2 Student Research Committee, Qazvin University of Medical Sciences, Qazvin, Iran; 3 Department of Biostatics, Qazvin University of Medical Sciences, Qazvin, Iran

**Keywords:** *Stenotrophomonas maltophilia*, Biocide-resistance, Antibiotic resistance, Nosocomial infection, Biofilm

## Abstract

**Background:**

The overuse of biocides in healthcare-facilities poses risk for emergence and spread of antibiotic resistance among nosocomial pathogens. Hospital-acquired infections due to *S. maltophilia* have been increased in the recent years and with its various resistance mechanisms contribute to patient morbidity and mortality in hospitals. The current study aimed to evaluate the susceptibility of biofilm-producing and non-producing *S. maltophilia* clinical isolates to five commonly used hospital biocides, alone and in combination with EDTA to examine the synergistic effect of combining EDTA on the bactericidal activity of them by microbroth dilution method. As well as the frequency of efflux genes encoding resistance to biocides among isolates. This study also intended to assess the effect of exposure of *S. maltophilia* isolates to sub-inhibitory concentrations of sodium hypochlorite upon the antimicrobial susceptibility patterns.

**Results:**

Based on minimum inhibitory and bactericidal concentrations of biocides sodium hypochlorite 5% (w/v) and ethyl alcohol 70% (v/v) were the strongest and weakest biocides against *S. maltophilia* isolates, respectively. The combination of EDTA with biocides significantly increased the effectiveness of the studied biocides. Exposure to sub-inhibitory concentration of sodium hypochlorite showed a significant change in the susceptibility of isolates towards ceftazidime (*p* = 0.019), ticarcillin/clavulanate (*p* = 0.009), and chloramphenicol (*p* = 0.028). As well as among the isolates examined, 94 (95%) were able to produce biofilm. The frequency of *sugE1* resistance genes was found in 90.7% of our clinical *S. maltophilia* isolates. None of the isolates carried *qacE* and *qacEΔ1* gene.

**Conclusions:**

The current study recommended that using the mixture of biocides with EDTA can be effective in reducing nosocomial infections. Also, this study demonstrated that exposure to sub-inhibitory concentrations of sodium hypochlorite leads to reduced antibiotic susceptibility and development of multidrug-resistant *S. maltophilia* strains.

**Supplementary Information:**

The online version contains supplementary material available at 10.1186/s12866-022-02664-1.

## Background

Hospital-acquired infections are recognized as one of the problematic challenges for infection control worldwide [1]. Healthcare facilities and environments provide an ideal reservoir for the growth, colonization, and proliferation of pathogenic organisms [2, 3]. *Stenotrophomonas maltophilia*, formerly known as *Pseudomonas maltophilia* or *Xanthomonas maltophilia* is a common cause of hospital-acquired infection [4]. Despite of limited pathogenicity of this bacterium*, S. maltophilia* is known as one of the leading antibiotic-resistant pathogens and is associated with a variety of life-threatening nosocomial infections including pneumonia, bacteremia, endocarditis, respiratory and urinary tract infection, wound and soft tissue infections in hospitalized or immunocompromised patients due to its intrinsic resistance nature and acquiring resistance of this bacterium against multiple antimicrobial agents and biocides through plasmids, transposons, integrons, and limited therapeutic options [4–6]. Additionally, the ability to adhere and develop biofilm both on biotic and abiotic surfaces and survive in adverse environmental conditions, enables *S. maltophilia* to causes infection and contributes to chronic infections. Resistance to antimicrobial agents is the most important property of biofilm development [7, 8].

The increasing prevalence of biocide resistance and the potential for cross-resistance to some antibiotics is one of the global health threats and result in hospital-acquired infections and ineffective treatments [9, 10]. The development of resistance to biocides by bacteria is a public health hazard [10]. Biocides including antiseptics and disinfectants with proper use, are an essential part of public health and have a crucial role in preventing colonization and infection and controlling pathogenic bacteria in the hospital. The effectiveness of biocides depends on several factors such as concentration, the status of bacteria (biofilm or planktonic), and presence of genes conferring resistance to biocides [10]. Based on mentioned points, there is a rational concern that the misuse of biocides such as high or inadequate concentrations, and frequent exposure to sub-inhibitory concentrations (concentrations below those required to arrest growth) could select for strains that are tolerant to and could render them ineffective and may contribute to antibiotic resistance and leads to the development of multi-drug resistant (MDR) strains [11]. One of the well-known mechanisms responsible for resistance to biocides is the expression of efflux systems involving *qac* genes (*qacE* and its mutant form *qacE∆1*) and *sugE* gene [12]. The *sugE* gene along with *qacE* and *qacEΔ1* genes which are members of small multidrug resistance (SMR) family conferring resistance to quaternary ammonium compounds (QACs) [12–14]. Increasing expression of efflux pump genes results in MDR isolates [15]. The co-resistance and cross-resistance to biocides and antibiotics could be relevant to genes encoding resistance to biocide horizontally transferred mobile genetic elements that also carry antibiotic resistance genes [16].

On the other hand, reports indicate that application of chelating agents such as ethylenediaminetetraacetic acid (EDTA) can often enhance biocidal activity of antimicrobial agents. EDTA is a well-established metal chelator that disrupt the lipopolysaccharide structure in the outer membrane of Gram-negative bacteria and can change the outer membrane permeability; as a result, it becomes more permeable and sensitive to antimicrobial agent [17, 18]. EDTA has been known as a ‘potentiator’ of the efficacy of other antimicrobial agents. EDTA can prevent and reduce the risk of bacterial biofilm formation and colonization by disrupting biofilm due to its ability to cations sequestering (Mg2+, Ca2+, and Fe3) [19–21]. Accordingly, the combination of biocides with EDTA leads to synergistic effects and could be prove useful in preventing of transmission and emergence of resistant strains, reduction of nosocomial infections, consequently improve therapy efficacy [18, 19, 22].

Since as, few published studies are available to assessing reduced susceptibility to biocides than antibiotics and also about antibiotic resistance induced by increased resistance to biocides against *S. maltophilia*. We aimed to evaluate the susceptibility of *S. maltophilia* isolates to five commonly used biocides including ethyl alcohol 70%, sodium hypochlorite 5%, dettol 4.8%, sayasept HP 2%, chlorhexidine 2% in two steps, with or without EDTA to examine the impact of EDTA on the bactericidal activity of the studied biocides. Another objective was to investigate the effect of exposure to sub-inhibitory concentrations of the sodium hypochlorite on antimicrobial susceptibility patterns of *S. maltophilia* clinical isolates in Iran. Also, the present study was also aimed to evaluate the biofilm formation capacity of isolates by microtiter plate assay, as well as the detection of the presence of efflux pump genes (*qacE, qacEΔ1, and sugE1*) by conventional PCR technique among them. Undoubtedly, the results of this study and understanding the susceptibility of *S. maltophilia* to biocides and its association with antibiotic resistance will contribute to the control of this bacterium in hospitals and aid in the prevention of nosocomial infection.

## Results

### Description of clinical isolates

Biochemical tests and the presence of a 638-*bp* fragment of *23S rRNA gene* in 97 test isolates confirmed their identity as *S. maltophilia* [23]. Out of 97 isolates, 55 (56.7%) and 7 (7.2%) isolates were collected from Shariati and Children medical center hospitals affiliated to Tehran University of Medical Sciences (TUMS) in Tehran and 25 (25.8%) isolates from Velayat, 8 (8.2%) from Bouali and Ghods with 2 (2.1%) isolates from admitted patients in hospitals affiliated to Qazvin Medical University (QUMS) in Qazvin. Among them, 59 (60.8%) isolates were from males and 38 (39.2%) isolates were from females (male: female ratio = 1.5). The range of patients’ age was from 2 days to 85 years and 9 (9.3%) of the isolates were recovered from infants and 3 cases of whom were from infants less than 1 month of age (< 1). Blood was the major source of isolates (*n* = 82; 84.5%) and the remaining isolates were recovered from tracheal aspirate (*n* = 6; 6.2%) followed by bronchoalveolar lavage (*n* = 3; 3.1%), ocular discharge (*n* = 2; 2.1%), sputum (*n* = 2; 2.1%), urine (*n* = 1; 1%), and ascites fluid (*n* = 1; 1%) (Table 1). The majority of *S. maltophilia* isolates (*n* = 57; 58.8%) were obtained from patients admitted to emergency wards.

**Table 1:** Occurrence of *S. maltophilia* biofilm in relation to clinical source

Clinical source (no. of isolates)	No. (%) of isolates with biofilm phenotype			
	None	Weak	Intermediate	Strong
Blood culture (82)	4 (4.1)	3 (3.09)	39 (40.2)	36 (37.1)
Urine culture (1)	–	–	–	1 (1.03)
Trachea culture (6)	–	2 (2.06)	1 (1.03)	3 (3.09)
BAL (3)	–	1 (1.03)	–	2 (2.06)
Eye discharge culture (2)	–	–	–	2 (2.06)
Sputum (2)	–	1 (1.03)	1 (1.03)	–
Ascites (1)	–	–	–	1 (1.03)
Total (97)	4 (4.1)	7 (7.2)	41 (42.3)	45 (46.4)

*BAL* bronchoalveolar lavage

### Antimicrobial susceptibility testing

The antimicrobial susceptibility patterns of the isolates, using the disk diffusion method, before treatment with biocides are shown in Table 2. Of the 97 *S. maltophilia* isolates, 25 (25.8%) were multidrug-resistant and 10 (10.3%) isolates were extensively drug-resistant according to CLSI interpretive criteria [24]. *S. maltophilia* isolate was defined as multidrug-resistant (MDR) and Extensively drug-resistant (XDR), if it exhibited non-susceptibility to at least one agent in three or more and at least one agent in all but two or fewer antimicrobial categories including β-lactam/β-lactamase inhibitor combinations, sulfonamides, fluoroquinolones, chloramphenicol, cephalosporins, tetracyclines, and glycylcyclines, respectively [25]. As shown in Table 2, among *S. maltophilia* isolates examined, all of them were highly resistant (100%) to imipenem and meropenem, and 10 (10.3%) isolates showed resistance to trimethoprim/ sulfamethoxazole (TMP–SXT) and 4 (4.1%) indicated intermediate resistance. Levofloxacin, minocycline, and tigecycline exhibited the highest susceptibility of 97.9, 88.7, and 86.6%, respectively. The susceptibility rates of isolates to other antimicrobials by disk diffusion were as follows: chloramphenicol (60.8%); ticarcillin/clavulanate (39.2%); ceftazidime (23.7%).

**Table 2:** Antimicrobial susceptibility profiles of *S. maltophilia* before and after exposure to sub-inhibitory concentrations of sodium hypochlorite

Antibiotic	No. of isolates of ***S. maltophilia*** (%)						***p***-value
	Before exposure with sodium hypochlorite			After exposure with sodium hypochlorite			
	Sensitive	Intermediate	Resistance	Sensitive	Intermediate	Resistance	
Trimethoprim-sulfamethoxazole	83 (85.6)	4 (4.1)	10 (10.3)	83 (85.6)	4 (4.1)	10 (10.3)	1
Meropenem	0 (0)	0 (0)	97 (100)	0 (0)	0 (0)	97 (100)	1
Imipenem	0 (0)	0 (0)	97 (100)	0 (0)	0 (0)	97 (100)	1
Levofloxacin	95 (97.9)	2 (2.1)	0 (0)	95 (97.9)	2 (2.1)	0 (0)	1
Minocycline	86 (88.7)	10 (10.3)	1 (1)	86 (88.7)	10 (10.3)	1 (1)	1
Ceftazidime	23 (23.7)	12 (12.4)	62 (63.9)	17 (17.5)	17 (17.5)	63 (64.9)	0.019
Tigecycline	84 (86.6)	12 (12.4)	1 (1)	84 (86.6)	12 (12.4)	1 (1)	1
Chloramphenicol	59 (60.8)	32 (33)	6 (6.2)	50 (51.5)	35 (36.1)	12 (12.4)	0.028
Ticarcillin/clavulanate	38 (39.2)	34 (35.1)	25 (25.8)	25 (25.8)	37 (38.1)	35 (38.1)	0.009

### Determination of minimum inhibitory and bactericidal concentrations (MICs/MBCs) of biocides

The susceptibility of five biocides including sodium hypochlorite 5%, dettol 4.8%, ethyl alcohol 70%, sayasept-HP 2%, chlorhexidine 2% was tested against 97 *S. maltophilia* isolates using concentrations ranging from 2 to 512 μg/ml (50–0.19%). The obtained MIC and MBC results for all of the isolates are shown in Table 3. All of the tested biocides except ethyl alcohol, at MIC and MBC 1/2–1/8 had a complete inhibitory and lethal effect on the *S. maltophilia* isolates. As Table 3 shows, the MIC values of the biocides tested were quite variable and in the following ranges: from 64 to 512 μg/mL for sodium hypochlorite, 64 to 256 μg/mL for dettol, 32 to 256 μg/mL for chlorhexidine, 16 to 128 μg/mL for sayasept-HP and 8 to 128 μg/mL for ethyl alcohol. The isolates with sodium hypochlorite and dettol MICs of 128 μg/ml, sayasept-HP and chlorhexidine MICs of 64 μg/ml, and ethyl alcohol MICs of 32 μg/ml were observed often. The MBCs ranged from 64 to 512 μg/mL for sodium hypochlorite, 32–256 μg/mL for dettol and chlorhexidine, 16–128 μg/mL for sayasept-HP, and 8–32 μg/mL for ethyl alcohol in *S. maltophilia* isolates. The isolates with sodium hypochlorite and dettol MBCs of 64 μg/ml, sayasept-HP and chlorhexidine MBCs of 32 μg/ml, and ethyl alcohol MBCs of 16 μg/ml were observed most frequently.

**Table 3:** MIC and MBC values of biocides in presence and absence of EDTA17% determined for 97 *S. maltophilia* isolates

	Dilution rang tested N (%)									
Biocides	Serial dilution	1/2	1/4	1/8	1/16	1/32	1/64	1/128	1/256	1/512
Sodium hypochlorite 5%	Active ingredients	**2.5%**	**1.2%**	**0.62%**	**0.31%**	**0.15%**	**0.078%**	**0.039%**	**0.019%**	**0.0097%**
	MIC	–	–	–	–	–	24 (24.7%)	43 (44.3%)	27 (27.8%)	3 (3.1%)
	MIC+EDTA	–	–	–	–	4 (4.1%)	4 (4.1%)	4 (4.1%)	27 (27.8%)	58 (59.8%)
	MBC	–	–	–	–	–	45 (46.4%)	36 (37.1%)	15 (15.5%)	1 (1%)
	MBC + EDTA	–	–	–	–	5 (5.2%)	6 (6.2%)	15 (15.5%)	64 (66%)	7 (7.2%)
Dettol 4.8%	Active ingredients	**2.4%**	**1.2%**	**0.62%**	**0.31%**	**0.15%**	**0.075%**	**0.037%**	**0.018%**	**0.0093%**
	MIC	–	–	–	–	–	32 (33.0%)	50 (51.5%)	15 (15.5%)	–
	MIC+EDTA	–	–	–	–	–	8 (8.2%)	10 (10.3%)	48 (49.5%)	31(32%)
	MBC	–	–	–	–	11 (11.3%)	56 (57.7%)	28 (28.9%)	2 (2.1%)	–
	MBC + EDTA	–	–	–	–	3 (3.1%)	8 (8.2%)	27 (27.8%)	57 (58.8%)	2 (2.1%)
Chlorhexidine 2%	Active ingredients	**1%**	**0.5%**	**0.25%**	**0.125%**	**0.062%**	**0.031%**	**0.015%**	**0.0078%**	**0.0039%**
	MIC	–	–	–	–	17 (17.5%)	42 (43.3%)	37 (38.1%)	1 (1%)	–
	MIC+EDTA	–	–	–	2 (2.1%)	6 (6.2%)	1 (1%)	23 (23.7%)	62 (63.9%)	3 (3.1%)
	MBC	–	–	–	–	44 (45.4%)	41 (42.3%)	11 (11.3%)	1 (1%)	–
	MBC + EDTA	–	–	–	2 (2.1%)	6 (6.2%)	16 (16.5%)	45 (46.4%)	28 (28.9%)	–
Sayasept-HP 2%	Active ingredients	**1%**	**0.5%**	**0.25%**	**0.125%**	**0.062%**	**0.031%**	**0.015%**	**0.0078%**	**0.0039%**
	MIC	–	–	–	2 (2.1%)	38 (39.2%)	41 (42.3%)	16 (16.5%)	–	–
	MIC+EDTA	–	–	–	3 (3.1%)	5 (5.2%)	11 (11.3%)	41 (42.3%)	37 (38.1%)	–
	MBC	–	–	–	21 (21.6%)	49 (50.5%)	26 (26.8%)	1 (1%)	–	–
	MBC + EDTA	–	–	–	8 (8.2%)	3 (3.1%)	33 (34%)	50 (51.5%)	3 (3.1%)	–
Ethyl Alcohol 70%	Active ingredients	**35%**	**17.5%**	**8.78%**	**4.37%**	**2.18%**	**1.09%**	**0.54%**	**0.27%**	**0.13%**
	MIC	–	–	2 (2.1%)	38 (39.2%)	41 (42.3%)	15 (15.5%)	1 (1%)	–	–
	MIC+EDTA	–	–	–	7 (7.2%)	3 (3.1%)	32 (33%)	46 (47.4%)	9 (9.3%)	–
	MBC	–	–	35 (36.1%)	32 (33%)	30 (30.9%)	–	–	–	–
	MBC + EDTA	–	–	5 (5.2%)	6 (6.2%)	33 (34%)	38 (39.2%)	15 (15.5%)	–	–

The cells (−) means is not MIC and MBC for any of isolates

Two wells were growth (TSB + inoculation) and sterility (contained TSB alone) controls

The results of present study showed that among tested biocides, sodium hypochlorite 5% (the lowest MIC and MBC) and ethyl alcohol 70% (the highest MIC and MBC) were the strongest and weakest against *S. maltophilia* isolates, respectively. The most effective biocides were sodium hypochlorite 5%, dettol 4.8%, chlorhexidine 2%, saya sept-HP 2%, ethyl alcohol 70%, respectively. Also, the efficacy of the tested biocides was examined by using Rideal-Walker phenol Coefficient Test. As Table 4 shows, the phenol coefficients were calculated about 4.78, 0.6, 0.6, 0.15 and 0.038 for sodium hypochlorite, dettol, chlorhexidine, sayasept HP and ethyl alcohol, respectively. The resuls also, showed that the bactericidal efficiency of sodium hypochlorite was more than phenol and other disinfectants and had more lethality and ethyl alcohol had the lowest lethality.

**Table 4:** Rideal-walker phenol coefficient test

Min	2.5	5	7.5	10	Min	2.5	5	7.5	10	Min	2.5	5	7.5	10	Min	2.5	5	7.5	10	Min	2.5	5	7.5	10
Dilution					Dilution					Dilution					Dilution					Dilution				
phenol					phenol					phenol					phenol					phenol				
1/90	–	–	–	–	1/90	–	–	–	–	1/90	–	–	–	–	1/90	–	–	–	–	1/90	**–**	**–**	**–**	**–**
1/95	–	–	–	–	1/95	–	–	–	–	1/95	–	–	–	–	1/95	–	–	–	–	1/95	**–**	**–**	**–**	**–**
1/100	–	–	–	–	1/100	–	–	–	–	1/100	–	–	–	–	1/100	–	–	–	–	1/100	**–**	**–**	**–**	**–**
1/105	+	+	–	–	1/105	+	+	–	–	1/105	+	+	–	–	1/105	+	+	–	–	1/105	**+**	**+**	**–**	**–**
1/110	+	+	+	+	1/110	+	+	+	+	1/110	+	+	+	+	1/110	+	+	+	+	1/110	**+**	**+**	**+**	**+**
1/115	+	+	+	+	1/115	+	+	+	+	1/115	+	+	+	+	1/115	+	+	+	+	1/115	**+**	**+**	**+**	**+**
Ethyl alcohol 70%					Sodium hypochlorite 5%					Sayasept-HP 2%					Chlorhexidine 2%					Dettol 4.8%				
1/2	+	–	–	–	1/2	–	–	–	–	1/2	–	–	–	–	1/2	**–**	**–**	**–**	**–**	1/2	–	–	–	–
1/4	+	+	–	–	1/4	–	–	–	–	1/4	–	–	–	–	1/4	**–**	**–**	**–**	**–**	1/4	–	–	–	–
1/8	+	+	+	–	1/8	–	–	–	–	1/8	–	–	–	–	1/8	**–**	**–**	**–**	**–**	1/8	–	–	–	–
1/16	+	+	+	+	1/16	–	–	–	–	1/16	+	+	–	–	16-Jan	**–**	**–**	**–**	**–**	1/16	–	–	–	–
1/32	+	+	+	+	1/32	–	–	–	–	1/32	+	+	+	–	1/32	**+**	**–**	**–**	**–**	1/32	+	–	–	–
1/64	+	+	+	+	1/64	–	–	–	–	1/64	+	+	+	–	1/64	+	+	–	–	1/64	+	+	–	–
1/128	+	+	+	+	1/128	–	–	–	–	1/128	+	+	+	–	1/128	+	+	+	–	1/128	+	+	+	–
1/256	+	+	+	+	1/256	+	–	–	–	1/256	+	+	+	+	1/256	+	+	+	–	1/256	+	+	+	–
1/512	+	+	+	+	1/512	+	+	–	–	1/512	+	+	+	+	1/512	+	+	+	+	1/512	+	+	+	+
Phenol coefficient = 0.038					Phenol coefficient = 4.78					Phenol coefficient = 0. 15					Phenol coefficient = 0.6					Phenol coefficient = 0.6				

### Synergistic effect of biocides with EDTA treatment

The MIC and MBC of selected biocides in combination with EDTA 17% for all isolates were obtained by microbroth dilution method. As Table 3 shows, after adding EDTA, the MIC values of the mixture of biocides with EDTA were in the following ranges: from 32 to 512 μg/mL for sodium hypochlorite, 64 to 512 μg/mL for dettol, 16 to 512 μg/mL for chlorhexidine, 16 to 256 μg/mL for sayasept-HP and ethyl alcohol. The isolates with sodium hypochlorite MICs of 512 μg/ml, dettol MICs of 256 μg/ml, chlorhexidine MICs of 256 μg/ml, sayasept-HP and ethyl alcohol MICs of 128 μg/ml were observed often. Also, the changes in MBCs ranged from 32 to 512 μg/mL for sodium hypochlorite and dettol, 16–256 μg/mL for chlorhexidine and sayasept-HP, and 8–128 μg/mL for ethyl alcohol in *S. maltophilia* isolates. The isolates with sodium hypochlorite and dettol MBCs of 256 μg/ml, sayasept-HP and chlorhexidine MBCs of 128 μg/ml, and ethyl alcohol MBCs of 64 μg/ml were observed most frequently. Change of two-fold or higher in the MICs and MBCs of mixture of biocide with EDTA (reduction of concentration) in comparison with MIC and MBC values of biocide alone was considered as synergic effect of EDTA combination with biocides. The results of this study showed that inhibitory and lethality effects of the biocides were treated with EDTA relative to the effect of the biocides alone, were greater and showed a significant synergistic effect. Thus, our results indicated that adding EDTA increased the efficiency of all studied biocides. At the used concentrations, biocides without EDTA were efficient at higher concentrations (highest MIC and MBC) in comparison with biocides treated with EDTA, the effects of EDTA and biocides were additive. In our study showed that ethanol 70%, sodium hypochlorite 5% gave the best results when combined with EDTA than other of biocides (Table 3).

### Effect of exposure to sub-inhibitory concentrations of sodium hypochlorite on antimicrobial susceptibility pattern

The antimicrobial susceptibility of isolates was retested using disk diffusion only for antibiotic susceptible and susceptibility-intermediate isolates following exposure to sub-inhibitory concentrations of sodium hypochlorite. The obtained results before exposure to biocide were compared to those after exposure. The susceptibility patterns of some isolates either changed from susceptible to susceptibility-intermediate and resistant and from susceptibility-intermediate to resistant. Exposure to the sub-inhibitory concentration of sodium hypochlorite showed a significant change in the susceptibility of isolates towards ceftazidime (*p* = 0.019), ticarcillin/clavulanate (*p* = 0.009), and chloramphenicol (*p* = 0.028), which was susceptible or susceptibility-intermediate to them before exposure, whereas isolates did not show any difference in the susceptibility patterns of the other antibiotics upon exposure to sub-MICs of sodium hypochlorite. The isolates were categorized in MDR or XDR in the same manner as described in antimicrobial susceptibility testing. Exposure to the sub-inhibitory concentration of sodium hypochlorite showed a significant increase (*p* = 0.014) in the frequency of MDR and XDR *S. maltophilia* isolates. Our results showed that 4 isolates were exited from sensitive or intermediate antibiotic-categorize and categorized as MDR, and also 1 isolate became XDR. Collectively, 29 (29.9%) were multidrug-resistant and 11 (11.3%) isolates were extensively drug-resistant. Table 2 summarizes the susceptibility results tested before and after exposure with sub-MICs of sodium hypochlorite.

### Biofilm formation

The ability to develop biofilm varied greatly among the *S. maltophilia* isolates. Biofilm phenotypes accounted for 93 out of 97 isolates (95.9%) (Table 1). As Table 1 indicates, the results of the biofilm formation microtiter assay showed that 45 (46.4%) of isolates were strong biofilm producers, 41 isolates (42.3.%) were moderate biofilm producers and 7 isolates (7.2%) were weak biofilm-producers; whereas, 4 isolates (4.1%) did not form biofilm. Also, in the present study statistical analysis (Table 5) to evaluate the association between antibiotic resistance and biofilm production showed that between antibiotic resistance of ceftazidime (*p* = 0.049), ticarcillin-clavulanic acid (*p* = 0.001), and biofilm production was found to be statistically significant. This finding was not seen with other antibiotics.

**Table 5:** Association between antibiotic resistance and biofilm-forming ability of *S. maltophilia* isolates

Antibiotic	No. (%) of non-susceptible isolates	Biofilm formation ability				***p***-value
		Strong (***n*** = 45)	Intermediate (***n*** = 41)	Weak (***n*** = 7)	None (***n*** = 4)	
Trimethoprim/sulfamethoxazole	14 (14.4)	9	5	0	0	0.88
Imipenem	97 (100)	45	41	7	4	–
Meropenem	97 (100)	45	41	7	4	–
Levofloxacin	2 (2.1)	1	1	0	0	1.00
Minocycline	11 (11.3)	5	6	0	0	0.93
Ticarcillin/clavulanate	59 (60.9)	37	20	1	1	0.001
Tigecycline	13 (13.4)	9	4	0	0	0.34
Ceftazidime	74 (76.3)	40	26	4	0	0.049
Chloramphenicol	38 (39.2)	22	16	0	0	0.189

### PCR-based genotyping for *qacE, qacEΔ1*and *sug-E1*

**Fig. 1:**
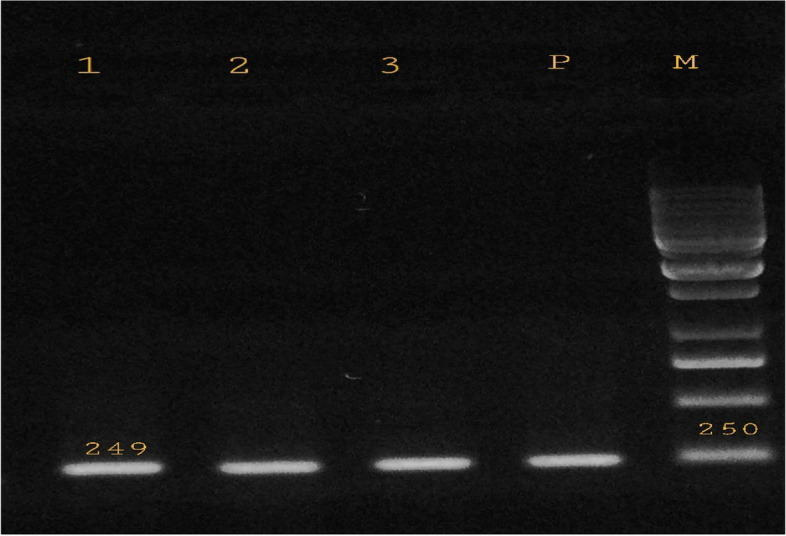
Gel electrophoresis of the PCR amplified products of *sugE1* gene for the *S. maltophilia* isolates with 249 bp amplification fragment. Lane M: DNA size marker - Lane P: positive control - Lane 1–3: *sugE1* positive isolates

PCR screening showed that among the 97 isolates tested, *sugE1* gene that confers resistance to biocides was present in 88 (90.7%) isolates (Fig. 1). Whereas, the *qacE* and *qacEΔ1* genes were not detected in any of the isolates. The nucleotide sequence of the *sugE1* gene was submitted to the GenBank database under accession number MZ503513.

## Discussion

Biocides with proper use, have a crucial role in preventing colonization and infection of pathogenic microorganisms and cuts off infection routes. Biocides including antiseptic and disinfectant have various efficacies depending on their use and the target microorganism [26]. The overuse and suboptimal concentrations of biocides used in hospitals for infection control might contribute to increased MICs and MBCs of them and leads to the development of resistance to biocides and multi-drug resistant strains [11]. Cross-resistance between antibiotics and biocides may occur via various and common mechanisms between them such as efflux pump systems, changes in permeability, and biofilm formation [27]. Since, the increasing emergence of bacteria with reduced susceptibility to biocides and the possible linkage between biocide and antibiotic resistance is a newly important concern [28–30], in this study we attempted to examine the bactericidal efficacy of five biocides against clinical isolates of *S. maltophilia* which can cause healthcare-associated infections (HAIs) and contribute to increase in patient morbidity and mortality [4]. The susceptibility to biocides was determined by comparing the MIC and MBC values against *S. maltophilia* isolates*.* Since there are no established breakpoints available for MICs and MBCs of biocides for defining resistance and sensitivity to biocides against bacteria, we tested twofold serial dilutions from 50 to 0.19% concentration of each biocide. In the present study, the biocides selected for susceptibility testing were sodium hypochlorite 5%, dettol 4.8%, chlorhexidine2%, sayasept-HP 2%, ethyl alcohol 70%, because they are widely used as antiseptic and disinfectant in healthcare facilities in Iran. Given that, there aren’t any criteria for categorization of bacteria as susceptible or resistant to biocides, it is not correct to consider bacteria that grow in low concentrations of biocides as resistant, this must be considered as increasing MIC value or reducing susceptibility to biocides according to inhibitory concentration of biocides values [31]. In our study evaluation of the susceptibility patterns from the isolates to biocides showed sensitivity to all selected biocides in all range of tested concentrations (1/2, 1/4, 1/8, 1/16, 1/32, 1/64, 1/128, 1/256, and 1/512). Sodium hypochlorite was the compound which showed the highest inhibition or lethal effect. Generally, among tested biocides, sodium hypochlorite 5% (w/v) (the lowest MIC and MBC) and ethyl alcohol 70% (v/v) (the highest MIC and MBC) were the strongest and weakest against *S. maltophilia* isolates, respectively. Overall, in the present study based on the minimum inhibitory and bactericidal concentration values, the most effective biocides were Sodium hypochlorite 5%, Dettol 4.8%, Chlorhexidine 2%, Saya sept-HP 2%, Ethyl alcohol 70%, respectively. In a study conducted by Bouzada et al. and other studies on the sodium hypochlorite, it was showed that sodium hypochlorite was more effective than quaternary ammonium compounds against bacteria which were consistent with our finding that, sodium hypochlorite was more effective than sayasept (fifth-generation of quaternary ammonium compounds) [32–34].

In the recent years combined usage of antibacterial agents such as antibiotics and other antimicrobial agents with EDTA has gained interest and broadly studied because it often leads to synergistic effects and could be useful to overcome problems with the development of resistance [20, 35]. Our results indicated that EDTA has a significant additive effect in the efficacy of studied biocides and result in increasing inhibitory and lethality power of biocides. Due to the observation of potentiation or synergy of biocides with EDTA, usage of these agents recommended and can be continued. The results of other studies against other bacteria confirm the results of our work [18, 36]. The finding of a study that conducted to evaluate the interference of EDTA in the antibacterial ability of sodium hypochlorite, showed that sodium hypochlorite was able to exert its full bactericidal action when added simultaneously with EDTA to a bacterial suspension which are consistent with present study [37]. Stevens et al. evaluated the inhibitory activity of nisin in combination with disodium EDTA against several *Salmonella* species and other selected gram-negative bacteria, the results showed that treatment with nisin or EDTA simultaneously decreased the growth of them while, EDTA or nisin alone produced no significant inhibition [38].

Some previous studies have demonstrated that antibiotic resistance can be induced by sub-MICs concentrations of biocide [33]. The effect of biocides on antimicrobial susceptibility in bacteria and the development of antibiotics-resistant healthcare-acquired microorganisms after treatment with sub-inhibitory concentrations of different biocides on surviving bacteria has been measured and confirmed [33, 39]. The reason for choosing sodium hypochlorite to examine the effect of sub-inhibitory on *S. maltophilia* strain in this study was the wide use of this disinfectant in the country for disinfection of surfaces and instruments. To our knowledge, no previous studies have investigated the effect of exposure to biocides on susceptibility patterns of *S. maltophilia* strains. Our finding demonstrated that *S. maltophilia* isolates could yield resistance toward antibiotics after overnight incubation with sodium hypochlorite, a statistically significant change was observed in susceptibility patterns of ceftazidime (*p* = 0.019), ticarcillin/clavulanate (*p* = 0.009), and chloramphenicol (*p* = 0.028). Notably, the number of multidrug-resistant *S. maltophilia* isolates has been shown a statistically significant increase (*p* = 0.014), in comparison to before exposure to the biocide. The results of our study, together with previous studies, suggest that exposure to the sub-inhibitory concentrations of various biocides can induce antibiotic resistance in the isolates [18, 33, 39–42].

Biofilms are associated with 65% of hospital-acquired infections [43]. Reports suggesting that biofilm formation is an important mechanism for resistance to antibiotics and biocides by *S. maltophilia* [44]*.* Here, we observed that all but four isolates investigated were biofilm-producers, although with different biofilm-forming abilities. The prevalence of *S. maltophilia* isolates able to develop biofilm in our study (95.9%) was like that (88.7–100%) in previous reports in Iran and Europe [6, 23, 43, 45]. Also, the present study examined the association between antibiotic resistance and potential of biofilm formation, these results demonstrated that there is a significant association between the potential of biofilm formation and resistance to ceftazidime (*p* = 0.049), and ticarcillin/clavulanate (*p* = 0.001) in *S. maltophilia*, which was consistent with the report by Sun et al. and the other study [23, 46]. From the results of our study, it can be concluded that strong and intermediate biofilm-producing strains have higher antibiotic and biocide resistance and need higher concentration (MIC and MBC) of biocides for killing of isolates.

The *sugE1* gene along with *qacE* and *qacEΔ1* genes which are members of small multidrug resistance (SMR) protein is also being known as a quaternary ammonium compound (QAC) resistance determinant [14, 47, 48]. As far as we know, a limited number of biocides resistance gene studies have involved clinical *S. maltophilia* isolates. In our study *qacE* and *qacEΔ1* genes were not detected in any isolates. In contrast, our findings have demonstrated high levels of presence of *sugE* gene (90%) in clinical isolates of *S. maltophilia.* C. wang et al. found that 2 out of 19 (10.5%) *S. maltophilia* isolates carried *qacΔE1*gene [49]. In a study conducted by Kücken et al. *qacEΔ1* and *qacE* genes were not found in any isolate out of 13 *S. maltophilia* isolates [12]. In the present study, due to the high prevalence of *sugE1* gene, there was no association between the presence or absence of this gene and resistance to the tested biocides (increased MICs and MBCs) against *S. maltophilia* isolates.

From the comparison between the obtained results, it can be concluded that bacterial antibiotic resistance is not necessarily a reason for resistance to biocides. In fact, a biocide can have a similar effect on an antibiotic-sensitive or resistant bacterium and the presence of biocides resistance gene and biofilm are effective in this regard. And also, the present study showed that as long as biocides are used in proper concentrations, they can prevent the growth and development of multi-drug resistant isolates. Whereas using suboptimal concentrations and exposure to sub-inhibitory concentrations of biocides such as sodium hypochlorite result in reduced antibiotic susceptibility and cross-resistance. The development of antibiotic-resistant *S. maltophilia* strains which can cause detrimental effects and increase nosocomial infections. Monitoring quality of hospital routine cleaning services or staff and bacteria susceptibility to antibiotic and biocides may useful in the management of nosocomial infections. The rotational use of different biocides is recommended to avoid the evolution of resistance or selection of resistant strains in the hospital environment.

## Conclusion

In conclusion, our study demonstrated that *sugE1* gene was commonly present among clinical *S. maltophilia*. There was no significant association between the presence or absence of *sugE* gene and increased MICs and MBCs observed in *S. maltophilia* isolates. Our results showed that the addition of EDTA significantly increased the efficacy of studied biocides and it is recommended to combine the usage of antiseptic and disinfectant with EDTA to increase potency and efficacy of them. This study showed that exposure to sub-inhibitory concentrations of sodium hypochlorite leads to reduced antibiotic susceptibility and the development of multidrug-resistant *S. maltophilia* strains. This study also demonstrated that although biofilm-forming capacity was highly conserved among clinical strains of *S. maltophilia*, there was a significant difference in phenotype among them. Consequently, the use of proper bactericidal concentrations of different biocides aid in the prevention and controlling the outbreak of nosocomial infections caused by multi-drug resistant bacteria such as *S. maltophilia.* This study emphasizes the need for using optimal concentrations of biocides and also recommends a large-scale study to evaluate reduced susceptibility to biocides of nosocomial pathogens.

## Materials and methods

### Isolation and identification

A total of 105 clinical isolates as *S. maltophilia* were collected in the present study during the period between September 2019 and March 2020 at five tertiary-care hospitals in Iran (Shariati, Children medical center affiliated to Tehran University of Medical Sciences and Bouali, Ghods, Velayat affiliated to Qazvin University of Medical Sciences. All of the isolates were identified using standard microbiological and biochemical methods such as Gram stain, catalase and oxidase tests, motility, oxidative or fermentative metabolism, deoxyribonuclease test agar (DNase), triple sugar iron agar (TSI), lysine decarboxylase and esculin hydrolysis (Merck, Germany) according to diagnostic microbiology textbooks manual such as Mahon and Baily and Scott [50, 51]. Genomic DNA was extracted from a single colony of each isolate with high pure PCR Template Preparation Kit (Roche company, Germany and Lot.No.21538900). The quality and quantity of extracted DNA were evaluated using the Nanodrop instrument and gel electrophoresis (Termo Scientifc, Waltham, MA, USA). All isolates were reconfirmed genotypically as *S. maltophilia* by PCR with specific primers illustrated in Table 6 to amplify a 638-bp fragment of the *23S rRNA* gene. All isolated were stored at − 20 °C in trypticase soy broth (TSB; Merck, Germany) supplemented with 15% glycerol for further analysis. *Pseudomonas aeruginosa* ATCC 27853 and *S. maltophilia* ATCC 13637 were used as the quality control strains. A representative amplicon of *23S rRNA* gene was subjected to sequencing and the sequence was deposited in GenBank and assigned the accession no MZ468054.

**Table 6:** List of primers used in the study

Primer	Gene name	PCR Products (bp)	References	Annealing Temperature
Primer sequence (5′ → 3′)				
F- GAATATTGACCTGCTTCC R- GAGGTGATTAGGAGTG	*23srRNA*	638	[14]	52
F- TGCGTTCCTGGATCTATCTG R- GACGATGCCAATGCCTTC	*QacE*	206	In study	53
F- TTGTTATCGCAATAGTTG R- AATGGCTGTAATTATTGAC	*QacEΔ1*	202	In study	51
F-TGGATCTATTCTGTTGTTCGC R- CATCGGGCTGACCTGCTC	*Sug-E1*	249	In study	54

### Antimicrobial susceptibility testing

Antimicrobial susceptibility testing of *S. maltophilia* isolates against meropenem (10 mg), imipenem (10 mg), trimethoprim/sulfamethoxazole (1.25/23.75 mg), levofloxacin (5 mg), and minocycline (30 mg) (Mast Group Ltd., UK) was determined using Kirby-Bauer disc diffusion method according to the criteria of the Clinical and Laboratory Standards Institute (CLSI 2020) guidelines [52]. The critical breakpoints of ceftazidime (30 mg) and ticarcillin/clavulanate (75/10 mg) of *Pseudomonas aeruginosa* were used for interpretation of the results because no breakpoints for *S. maltophilia* were recommended by the CLSI. The results of chloramphenicol (30 mg) and tigecycline (15 mg) were interpreted according to the CLSI breakpoints of *Enterobacteriaceae* and the Food and Drug Administration (FDA), respectively. The *P. aeruginosa* ATCC 27853 and *S. maltophilia* ATCC 13637 were used for susceptibility testing. Due to the intrinsic resistance nature of *S. maltophilia* to carbapenems, susceptibility to meropenem and imipenem was also determined to confirm the identity of the isolates [5, 23, 53].

### Biocide’s susceptibility testing

During this study five commonly used antiseptics and disinfectants in hospitals for clinical items and bio-cleaning of instruments and surfaces were subjected to testing including: Ethyl Alcohol (70% v/v, Razi, Iran), Dettol (Chloroxylenol 4.8% w/v, British company Reckitt), Domestic Bleach (sodium hypochlorite 40 G/L, Golrang company, Iran), Chlorhexidine Digluconate (2% w/v, Sigma-Aldrich), Sayasept-HP 2% (Fifth-generate QACs, Behban chemistry company., Iran).

Susceptibility testing for all mentioned biocides was performed using broth microdilution method [54]. In brief, in the beginning, the *S. maltophilia* isolates were grown overnight on Muller Hinton agar (Merck, Germany) at 37 °C*. S. maltophilia* suspensions were adjusted to a turbidity equivalent to that of a 0.5 McFarland standard with sterilized saline solution and then diluted 0.01% (v/v). The wells 1 to 9 of a sterile 96-well plate were filled with 100 μl of trypticase soy broth (TSB). To well 1, 100 μl of tested biocides were added, upon mixing well, two-fold serial dilutions of biocides were done in TSB to yield the desired concentration ranging from 2 to 512 μg/mL, followed by 100 μl of each tested isolate (1.5 × 10^6^ CFU/mL) were inoculated to wells 1 through 9 to each well. Active ingredient of biocides based on serial dilution are available in Table 3. The wells 10 and 11 were growth (TSB + inoculation) and sterility (contained TSB alone) controls, respectively. The final concentration of each well was equal to 5× 10^5^ CFU/mL. MICs were examined visually after incubation at 37 °C for 24 h. The lowest concentration of the tested biocides that inhibited visible bacterial growth and didn’t show turbidity was reported as the minimum inhibitory concentration (MIC). To determine the minimum bactericidal concentration (MBC), 100 μL was withdrawn from each well without visible bacterial growth were cultured onto Muller Hinton agar plates and incubated overnight at 37 °C. The MIC and MBC of each biocide for all 97 strains of *S. maltophilia* were determined using this method. The efficacy of the biocides was examined by using Rideal-Walker phenol Coefficient Test (Table 4) [55].

### Investigation of biocides synergy with EDTA treatment

MIC and MBC values of the selected biocides with EDTA 17% were repeated and determined using the two-fold broth dilution method cross sterile 96-well plate as described above. Briefly, the selected biocides were mixed with the said substance one by one (50 μl biocide + 50 μl EDTA 17%) and placed at room temperature for 15 minutes. The dilution series were prepared and inoculated plates were then incubated overnight at 37 °C. After incubation, MIC and MBC were calculated with the new mixture for all isolates. The obtained results were compared with the previous results and its synergistic effect was examined [56].

### Effect of exposure to sub-inhibitory concentrations of sodium hypochlorite on antimicrobial susceptibility of the isolates

The effect of exposure to sub-inhibitory concentrations of sodium hypochlorite on antimicrobial susceptibility of *S. maltophilia* isolates was determined by comparing the antimicrobial susceptibility patterns of isolates before and after exposing to sub-inhibitory concentrations of sodium hypochlorite. For this goal, the antimicrobial susceptibility was retested by disk diffusion method for isolates that had grown in the highest concentration of sodium hypochlorite that still allowed bacterial growth (sub-inhibitory concentration). Briefly, 20 μl of the suspension were withdrawn from wells containing the highest concentration of sodium hypochlorite which still allows bacteria to grow (sub-MIC) and were aseptically transferred to the 5 ml sterile nutrient broth and were incubated at 37 °C until was adjusted to a turbidity equivalent to that of a 0.5 McFarland standard (4–6 h) to isolate the survived organisms. An antibiogram test for those survived isolates was performed. The suspension was evenly swabbed over the surface of Mueller Hinton agar plates and then the inoculated plates were incubated at 37 °C for 18–24 h. Diameters of the zone of inhibition around the discs were measured and the isolates were categorized as sensitive, intermediate and resistant according to the critical breakpoints of antibiotics in the same manner as described for antimicrobial susceptibility testing. The results were compared with the previous results [33].

### Biofilm formation assay

Biofilm assay was conducted in triplicate in 96-well flat-bottomed polystyrene microplates to evaluate the capacity of biofilm production in *S. maltophilia* isolates as described previously with some modifications [57]. Initially, the bacterial suspensions were prepared with an optical density (OD) of 0.1 were adjusted by using sterile trypticase soy broth (TSB) at 600 nm (OD600) with a spectrophotometer. Then, 200 μl of adjusted inoculums were transferred in triplicate into sterile 96-well flat-bottomed microplates and incubated overnight in a 37 °C. A series of triplet wells contained TSB alone (uninoculated broth) was considered as negative control to ensure the sterility during the execution of the experiment. The media were then removed by slightly tapping the plate and washed three times with phosphate-buffered saline (PBS: PH 7.2). Adherent biofilms were fixed with methanol for 15 min and dried at room temperature. Then, the biofilms within the wells were stained with 200 μl of the aqueous solution of 1% (w/v) crystal violet for 15 min. To remove the dye attached to the biofilm layers, the wells were rinsed three times with PBS and the plate was air-dried, biofilms were detached by adding 200 μl of 33% acetic acid into each well for 15 min. The optical absorbance (optical density) was measured at 570 nm (OD570, ODC570) using a microtiter plate reader (BioTek, Epoch, USA). The point to be noted is that all experiments were carried out in triplicate and repeated three times. Additionally, the optical density cut-off value (ODC) was established and defined as three standard deviations (S.D) above the mean OD of the negative control. (ODC = the average OD of the negative control + 3 × S.D. of the negative control). The isolates were classified into four groups based upon the strength of biofilm formation as follows [45]: no biofilm production (OD ≤ ODC); weak biofilm-producer (ODC < OD ≤ 2 × ODC); moderate biofilm-producer (2 × ODC < OD ≤ 4 × ODC); and strong biofilm-producer (ODC <4OD).

### Screening and detection of *qacE*, *qacE∆1* and *sugE1*

The presence of *qacE*, *qacE∆1*, and *sugE1* genes that confer resistance to biocides was examined using the primers shown in Table 6. PCRs were conducted on a thermal cycler (Applied Biosystems, USA) in 25 μl reaction volume containing 10 μL of 2X Master Mix RED (Ampliqon, Denmark), 1 μl of 10 pmol of each primer (Sinaclon Co; Tehran, Iran), 50 ng of template DNA and 6 μl of sterile distilled water. PCR conditions were performed under the following thermal conditions: pre-denaturation at 94 °C for 5 min; 30 cycles of DNA denaturation for 1 min at 94 °C; annealing at 51–54 °C, according to the primers for each gene (Table 6) for 25 s, extension for 50 s at 72 °C and a final extension at 72 °C for 7 min. All of the amplified products were separated by electrophoresis in 1.8% agarose gel stained with green viewer (Parstous, Mashhad. Iran). PCR experiment was run in triplicate (from the same sample) for all isolates tested. A representative PCR amplicon of each gene with the corresponding PCR primers was sequenced by Sanger technology to ensure the specific amplification. The sequenced data obtained was viewed in Chromas software and alignment were conducted using BLAST (http://www.ncbi.nlm.nih.gov/BLAST/).

### Statistical analysis

Data are expressed frequency and percent. Pearson chi-square or Fisher’s exact test was used to determine significant differences between proportions. The non-parametric Wilcoxon signed-rank test was performed to comparison of the antibiotics’ patterns before and after exposure of *S. maltophilia* isolates to sodium hypochlorite. The values *p* < 0.05 were considered statistically significant. Statistical analysis was done using SPSS version 16.0 statistical software (SPSS Inc., Chicago, IL, USA).

## Supplementary Information

**Additional file 1.**

## Data Availability

All data and materials are available upon request to corresponding author. The datasets generated and/or analysed during the current study are available in the GenBank repository. Gene data: *23S rRNA* sequence data: GenBank accession number MZ468054. *SugE1* sequence data: GenBank accession number MZ503513.

## Abbreviations

*S. maltophilia*: *stenotrophomonas maltophilia*
MDR: Multidrug-resistant
XDR: Extensively drug-resistant
MIC: Minimum inhibitory concentration
MBC: Minimum bactericidal concentration
EDTA: Ethylene- diamine-tetra acetic acid
QACs: Quaternary ammonium compounds
