# Supplementary material for: Evaluation of antibacterial activity of five biocides and the synergistic effect of biocide/EDTA combinations on biofilm-producing and non-producing Stenotrophomonas maltophilia strains isolated from clinical specimens in Iran
Source: BMC Microbiol. 2022 Oct 21;22:257. doi: 10.1186/s12866-022-02664-1 (PMC9585780; doi:10.1186/s12866-022-02664-1)

Gel electrophoresis of the PCR amplified product for detection of *sugE1* gene. *SugE1* gene (249bp)

The following cropped version of gel have included in the manuscript figure with DNA ladder 250 bp.

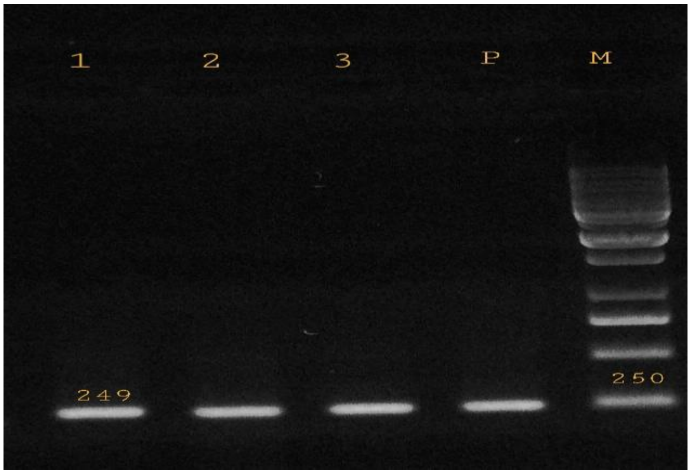

Fig1: Gel electrophoresis of the PCR amplified products of *sugE1* gene for the *S. maltophilia* isolates with 249 bp amplification fragment. Lane M: DNA size marker - Lane P: positive control - Lane 1-3: *sugE1* positive isolates

The original and unprocessed gel photograph of cropped figure in the manuscript with DNA ladder 250bp are shown below. We have cropped the image to improve the clarity and conciseness of presentation of our 4 samples.

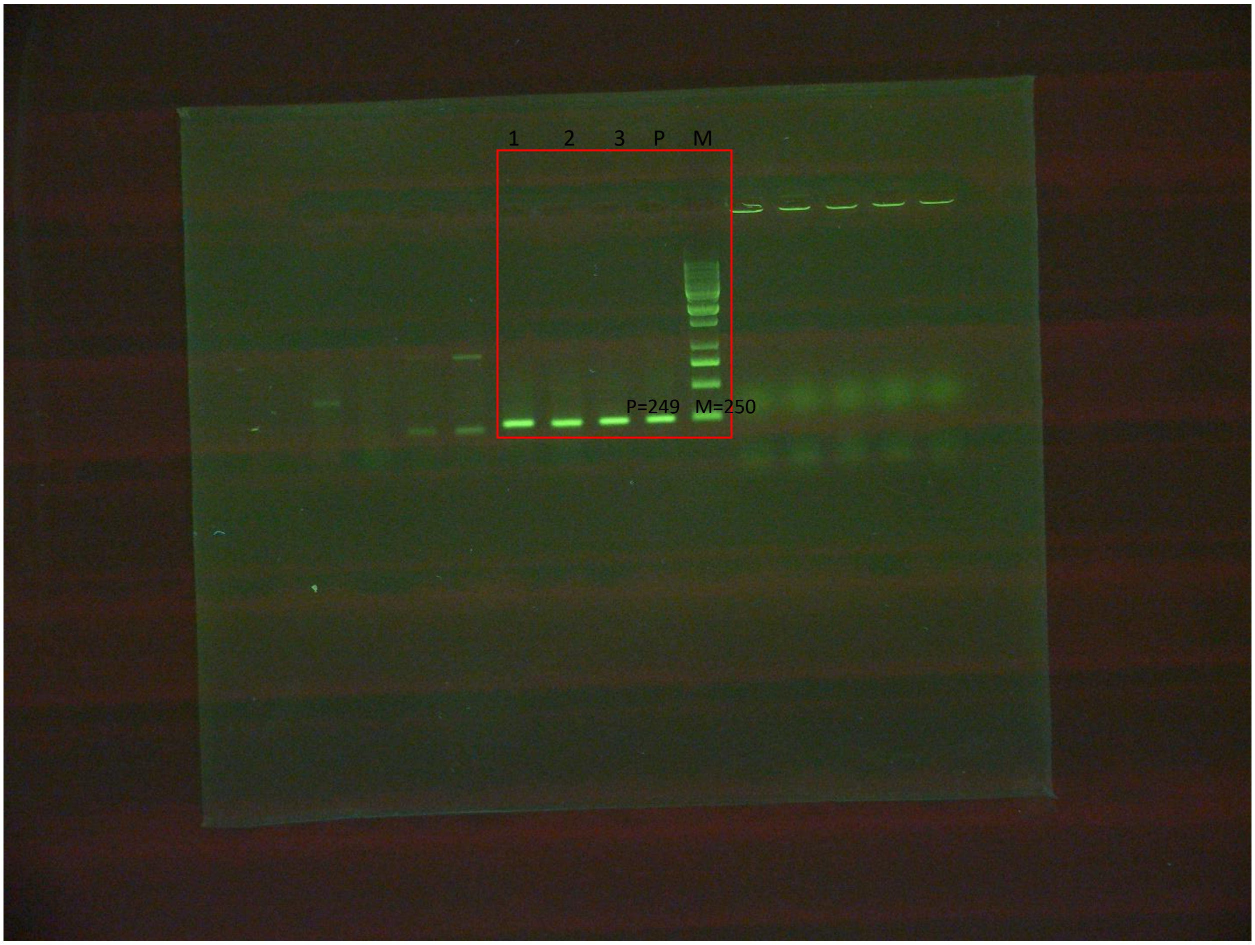

The cropped of original figure

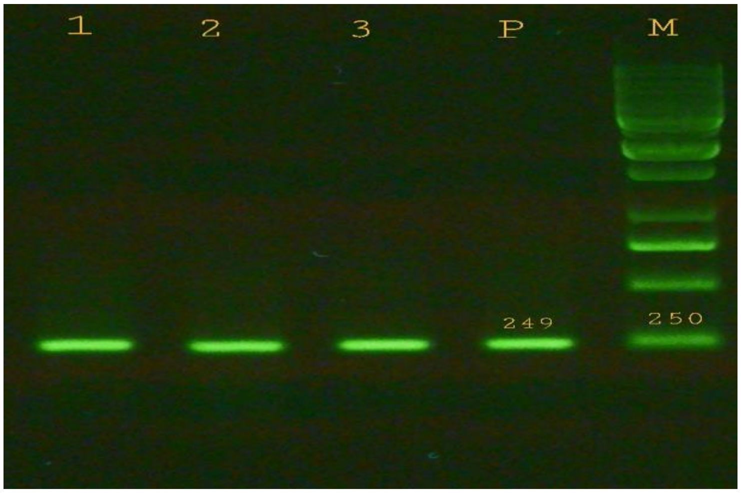

The following replicate of the PCR amplified product for detection of *sugE1* gene with DNA ladder 100bp have included in this file only to confirm our data. This gel photograph was not shown in the manuscript.

M= DNA ladder

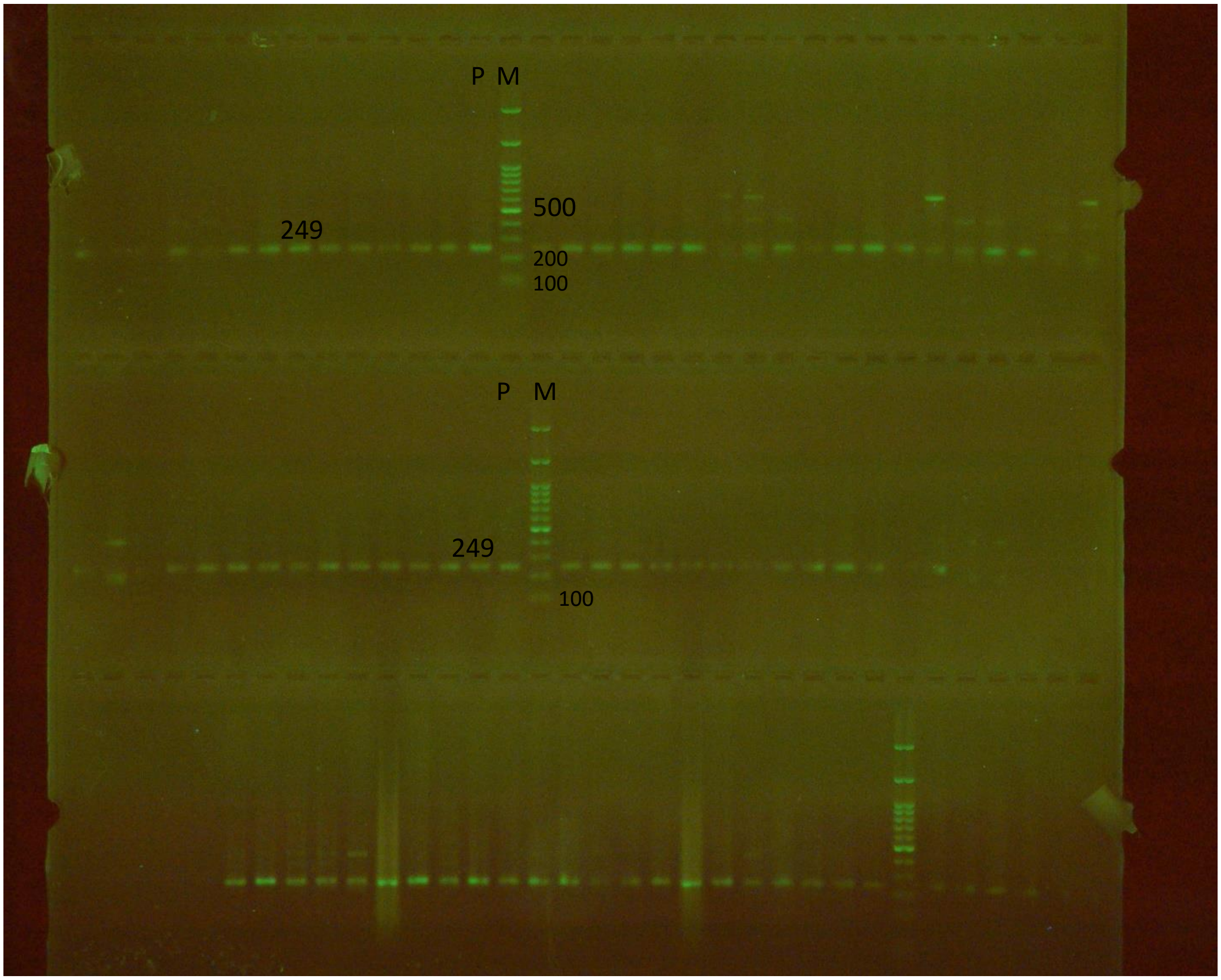

Supplement: Supplementary file 1 — Additional file 1. [file 12866_2022_2664_MOESM1_ESM.pdf]
